# PAIR: polymorphic Alu insertion recognition

**DOI:** 10.1186/1471-2105-13-S6-S7

**Published:** 2012-04-19

**Authors:** Jón Ingi Sveinbjörnsson, Bjarni V Halldórsson

**Affiliations:** 1 School of Science and Engineering, Reykjavik University, Reykjavík, 101, Iceland

## Abstract

**Background:**

Alu polymorphisms are some of the most common polymorphisms in the genome, yet few methods have been developed for their detection.

**Methods:**

We present algorithms to discover Alu polymorphisms using paired-end high throughput sequencing data from multiple individuals. We consider the problem of identifying sites containing polymorphic Alu insertions.

**Results:**

We give efficient and practical algorithms that detect polymorphic Alus, both those that are inserted with respect to the reference genome and those that are deleted. The algorithms have a linear time complexity and can be run on a standard desktop machine in a very short amount of time on top of the output of tools standard for sequencing analysis.

**Conclusions:**

In our simulated dataset we are able to locate 98.1% of Alus inserted with respect to the reference and 97.7% of Alus deleted, our simulations also show an excellent correlations between the deletions detected in parents and children. We further run our algorithms on publicly available data from the 1000 genomes project and find several thousand Alu polymorphisms in each individual.

## Introduction

We consider the problem of detecting polymorphic Alu insertions from DNA sequence reads using high throughput paired-end sequencing data.

Genomewide association studies (GWAS) proceed by identifying a number of individuals carrying a disease or a trait and comparing these individuals to those that do not or are not known to carry the disease/trait. Both sets of individuals are then genotyped for a large number of Single Nucleotide Polymorphism (SNP) genetic variants which are then tested for association to the disease/trait. GWAS have been able to successfully identify a very large number of polymorphism associated to disease (e.g. [1-3]). Studies using tens of thousands of individuals are becoming commonplace and are increasingly the norm in the association of genetic variants to disease [1-3].

Whole genome resequencing using next generation sequencers is rapidly becoming the sledgehammer of genomewide association studies. Increasingly, GWAS are done in conjunction with the sequencing of number of individuals [4,5] or alternatively using variants identified from the resequencing of a number of individuals [6]. Whole genome resequencing is preferable over SNP genotyping for association studies as it allows for the detection of all genomic variation and not only SNP variation. SNPs are the most abundant form of variation between two individuals. However, other forms of variation exist, such as inversions, copy-number variations, LINE (Long INterspersed Elements) and SINE (Short INterspersed Elements) elements, including Alu insertions.

Copy number variations, have been shown to be influential factors in many diseases [7], and a number of methods have been proposed for the detection of structural variants (e.g. [8-12]). Despite the fact that our computations indicate that the number of polymorphic Alu repeats carried by an individual are on a comparable scale to the number of copy number variations carried by an individual, apart from [13], no reliable methods have been specifically developed for detecting Alu repeats in multiple individuals. Polymorphic Alus are also known to be good markers for constructing phylogenetics of homonid evolution [14] and determing human diversity [15].

An Alu sequence is an approximately 300 basepair long sequence derived from 7SL RNA gene [16]. Alu repeats are SINE that occur frequently in the human genome, as well as in other genomes. The Alu sequence family has been propagated to more then one million copies in primate genomes over the last 65 million years. Alu repeats are the largest family of mobile elements in the human genome and the Alu family comprises more then 10% of the human genome. Most Alu repeats were inserted early in primate evolution, where it is estimated that there was approximately one new Alu insertion in every primate birth [17].

Almost all of the recently integrated human Alu elements belong to one of several small and closely related *young *Alu subfamilies, while other elements have been found to be largely orthologous to other primates. These largely human-specific AluY subfamilies represent approximately 0.5% of all the Alu repeats in the human genome. Our computations verify that AluY is the most polymorphic Alu family in our dataset.

The current rate of Alu insertion is estimated to be of the order of one Alu insertion in every 200 births [18]. Some members of these young Alu subfamilies have been inserted into the human genome so recently that they are polymorphic with respect to the presence or absence of insertion in different human genomes. Those relatively few elements that are present in the genomes of some individuals and absent from others are referred to as Alu-insertion polymorphisms. The primary goal of this paper is the discovery of these Alu insertion polymorphisms.

We give an algorithm targeted to finding Alu polymorphism from next generation paired-end sequencing data. In what follows we will start by giving our problem framework, followed by a description of our algorithms and finally we show some experimental results.

## Methods

### Problem framework

The input to our problem is a reference genome and a set of paired-end sequence reads from a set of individuals. The genome sequence of the reference individual is known and will be highly similar, but not identical, to the genome of the individual(s) being sequenced. Paired-end sequencing reads consist of a read of a fixed length, followed by a short spacing, followed by another read. The spacing between the two reads follows a probability distribution, *Y*. *Y *can be assumed to be known a priori or to be easily estimated from the sequence reads [19] (cf. Additional file 1 for the estimation of *Y*). The two reads are substrings of DNA sequence, with one read being read from the + strand and the other being read from the - strand. The fact that the two reads are read in opposite direction ensures that; If the location of one of the reads is known then the location of the *mate *(the other read) is also known, up to *Y*. The genome sequence of the individual(s) being sequenced is however not known a priori, but is highly similar to the reference genome. At some locations in the reference genome the genomes of the reference and the individual(s) being sequenced will diverge. Some of this divergence is due to the insertion of Alu polymorphisms. A mechanism exists for Alu sequences to insert themselves into a genome while no such direct mechanism is known to exist for Alu sequences to remove themselves from the genome. Once inserted, the sequence will exist in the sequence context where it was inserted.

When the polymorphic Alu is not contained in the reference, we consider the Alu to be inserted with respect to the reference. When the polymorphic Alu sequence is contained in the reference genome and some of the sequenced individuals we consider the Alu sequence to be deleted with respect to the reference, even though evolutionary the sequence most likely has been inserted.

The output of our algorithm is a set of locations in the genome where an Alu sequence is inserted in some individual(s) as well as the sequence reads of the individuals being studied for these insertions. As each individual contains two haplotypes a polymorphic Alu may be inserted on one, both or neither of these haplotypes.

We formulate four versions of the problem of identifying Alus, when the Alu sequences are inserted or deleted with respect to the reference genome, both for identifying these polymorphism on a single individual and on multiple individuals.

#### Problem 1

##### Single Individual Deleted Alu identification problem

**Input **A set of paired-end sequence reads from a single individual and a reference genome.

**Output **A list of locations in the genome where an Alu is deleted with respect to the reference genome.

#### Problem 2

##### Multiple Individual Deleted Alu identification problem

**Input **A set of paired-end sequence reads from multiple individuals and a reference genome.

**Output **A list of locations in the genome where there exists an individual with an Alu deleted with respect to the reference genome.

#### Problem 3

##### Single Individual Inserted Alu identification problem

**Input **A set of paired-end sequence reads from a single individual and a reference genome.

**Output **A list of locations in the genome where an Alu is inserted with respect to the reference genome.

#### Problem 4

##### Multiple Individual Inserted Alu identification problem

**Input **A set of paired-end sequence reads from multiple individuals and a reference genome.

**Output **A list of locations in the genome where there exists an individual with an Alu inserted with respect to the reference genome.

Following the identification of polymorphic regions we need to determine which individuals are polymorphic for each polymorphism.

#### Problem 5

##### Alu genotyping problem

**Input **A single location in the reference genome known to contain a polymorphic Alu. A set of individuals and a set of sequence reads for each individual.

**Output **For each individual, a genotype call, assigning the individual 0, 1 or 2 copies of the given Alu, representing an Alu on neither, one or both haplotypes.

We start by giving the common algorithmic framework for our algorithms and then proceed to giving algorithms for each of the problems in turn. We start by describing our approach for the detection of deleted regions in a single individual. We then extend this to recognizing deletions in multiple individuals simultaneously. We then show how these ideas can be extended to identifying inserted Alus, first in a single individual and finally in multiple individuals simultaneously.

### Algorithm framework

Our algorithms start by mapping the sequence reads to the reference genome and analyzing the output of such a mapping.

#### Alu Mate

We start by preprocesing the sequence reads to make them easier for manipulation. The initial step of our algorithm is to map the sequencing reads to the human reference genome build 37 (hg19) using the Burrows Wheeler Aligner (BWA) [20]. The program outputs a mapping of all sequence reads to the genome and also outputs whether there are alternate locations in the genome with sequence alignment. An underlying assumption is that most of the reads are long and accurate enough that they will only map to a single location on the genome. Technology where each paired end is 100 bases or greater with accuracy over 98% is readily available and in use [4,5]. In random DNA the probability of such reads mapping to multiple places on the genome is extremely low. Reads mapping to Alu sequences however will almost always have multiple places on the genome that have similar quality mapping. Unless its mate is mapped to a proximal location, we will not use the mapping of such reads as input to our algorithm, but rather label such reads as Alu reads. We further align each read to the set of known Alu families and label those that align well to the database as Alu reads. Most paired-end mates of Alu reads will map uniquely to the genome. We note that from the mapping of the paired-end it is easy to determine whether the Alu sequence should be to the left or the right of the mapped sequence.

A read pair is defined as *improper *if the two ends of the pair map to locations that are inconsistent with the read pair distance *Y*. We store all such improper pairs where one end is an Alu read and refer to the mate of those reads as *Alu mates*. Each of these read pairs either gives evidence of an Alu insertion or the read is improperly mapped or read. We label the Alu mate with an *r *if the mapped read is to the right of the Alu sequence and label them with a *l *if the mapped read is to the left of the Alu mapped read. The first step of our algorithm is to search for all Alu mates. At the same time we store the position and chromosome of the Alu mate, whether it is an *l *or an *r *read, to which Alu the Alu read mapped, to which Alu family that Alu belongs, where within the Alu the Alu read mapped and how many best matches to the reference genomes for the read where found by BWA. We term this algorithm *Alu mate *and we observe that it runs in time that is on the order of the number of reads.

Lemma 1

Algorithm Alu Mate runs in *O*(*n*_*r*_) time, where *n*_*r *_is the number of reads.

#### Analysis of mapped reads

The output of Alu mate is a mapping of sequence reads to the reference genome and an assignment of *l *and *r *read labels.

**Figure 1:**
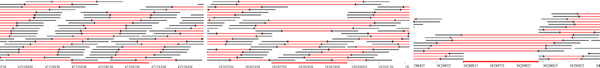
**Example of an Alu deletion**. Example of an Alu deletion. Arrows show read directions. Black arrows show normal mapping reads, red lines show the insert between them. The leftmost figure shows a normal individual, center an heterozygote and rightmost an individual homozygote for an Alu deletion. The location of the Alu is shown with a thick red line in the bottom of each figure.

Figure 1 shows the output of Alu mate and how it can be used to identify regions where an Alu is deleted with respect to the reference individual. Black arrows show the location and direction of the reads and the red lines show the insert between the reads. The location of the Alu is shown at the bottom of each figure. The leftmost figure shows an individual carrying the Alu on both of his chromosomes, notice that the distance between reads always follows the same distribution. The rightmost figure shows an individual that does not carry the Alu on either one of his chromosome (homozygote non-Alu), notice that the distance between the reads is longer for those reads overlapping the Alu and that no reads are mapped inside of the Alu. The center figure shows an individual heterozygote for the Alu.

**Figure 2:**
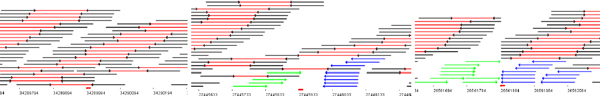
**Example of an Alu insertion**. Example of an Alu insertion. Arrows show read direction, black arrows show reads mapping normally, red lines show the insert between them, green arrows show *l *reads and blue arrow show *r *reads. The leftmost figure shows a normal individual, center an heterozygote and rightmost an individual homozygote for an Alu insertion. Red dot at the bottom of each figure shows the location of the Alu insertion.

Figure 2 shows the mapping the output of Alu mate and how it can be used to identify an Alu polymorphism. Black arrows show the reads and their direction. Red lines show the insert between the reads. Green arrows show *l *reads and blue arrows show *r *reads. Leftmost figure shows an individual carrying no copy of the Alu (homozygote non-Alu), notice the absence of *l *and *r *reads. The rightmost figure shows an individual homozygote for the Alu insertion, notice that the *l *reads occur to the left of the insertion and *r *reads to the right of the insertion and that no reads overlap the insertion. The center figure shows an individual carrying a single copy of the Alu (heterozyte).

### Detection of deleted Alus

We consider an Alu sequence deleted when it occurs in the reference assembly, but not in the individual(s) being sequenced. There are two primary signs of deletion, some of the reads will be split, containing one part from each side of the deletion. The second signal is that there are reads that have one end mapping to each of the two sides of the Alu being considered and a corresponding increase in their insert length. The distance between these reads, as measured with respect to the reference genome will be in expectation be longer than *Y *and should be distributed as *Y *+ *l*_*Alu*_, where *l*_*Alu*_, is the length of the deleted Alu. Detecting deleted Alus is considerably simpler than detecting inserted Alus, as the location of the Alu is known. For detecting Alu deletions we hence only need to consider locations that have been already annotated to contain Alus.

#### Genotyping deleted Alus

For each Alu annotated in the reference genome we determine the genotypes of the polymorphism of the individual. We let *Y*_*ϵ *_be the *ϵ *percentile of *Y *and *Y*_1-*ϵ *_be the 1 - *ϵ *percentile of *Y*, where *ϵ *is a small constant (0.005). At each annotated Alu we consider a window of size *Y*_1-*ϵ *_to the left and right of the estimated Alu.

We construct a set *T *consisting of all reads where both ends are in a window containing the Alu and *Y*_1-*ϵ *_to the left and right of the Alu. Here *l *and *r *are defined as before, r if the Alu sequence is to the right of the read and l if the Alu sequence is to the left. All *l *and *r *reads falling in that window are realigned to the Alu being considered. All reads where only one end maps inside the window and are not Alu mates are ignored.

We then compute the probability of observing the insert lengths in *T *given three different genotype models: Homozygote Alu, heterozygote and homozygote non Alu. We note that on chromosomes where there is an Alu sequence present then the reads mapping with one end inside of the Alu and one to the right of the Alu and the reads that map with one end to the left of the Alu and one inside of it will be independent of each other. On chromosomes where there is not an Alu sequence present the reads to the left and the right of the purported Alu location will be perfectly dependent. If our model is that reads are randomly sampled from the chromosome the reads can fulfill the criteria of belonging to *T *in one of three ways, each being equally likely; From the chromosome carrying the deletion, as a read pair mapping with one end inside the Alu and the other to the left of the Alu and as a read mapping with one end inside the Alu and the other to the right of the Alu. For the heterozygote case the probability that a read comes from the distribution *Y *is then $\frac{2}{3}$, while the probability of coming from *Z *= *Y *+ *l*_*Alu *_is $\frac{1}{3}$, where *l*_*Alu *_is the length of the Alu.

$$\begin{array}{c} \;P\left(data|HomoNonAlu\right)=P\left(D|0\right)=\underset{t\in T}{\prod }Y\left(t\right)\\ P\left(data|Hetero\right)=P\left(D|1\right)=\underset{t\in T}{\prod }\left(\frac{1}{3}Z\left(t\right)+\frac{2}{3}Y\left(t\right)\right)\\ \;\;P\left(data|HomoAlu\right)=P\left(D|2\right)=\underset{t\in T}{\prod }Z\left(t\right) \end{array}$$

#### Deleted Alus in Multiple Individuals

When considering multiple individuals simultaneously we can construct a likelihood ratio statistic for the occurance of the deletion. We let the individuals be labeled from 1 through *m*, *D*_*i *_be the set of sequence reads belonging to individual *i*. We let *f*_0 _be the frequency of the homozygote Alu carriers in the population, *f*_1 _be the frequency of the heterozygote and *f*_2 _be the frequency of the homozygote non-Alu. Then the joint likelihood of the data given an Alu deletion is:

$$\prod_{i=1}^{m}\left(f_{0}P\left(D_{i}|0\right)+f_{1}P\left(D_{i}|1\right)+f_{2}P\left(D_{i}|2\right)\right)$$

We apply a likelihood ratio test to test whether a deletion is significantly more likely than the model on the statistic

$$2\overset{m}{\underset{i=1}{\sum }}\text{log}\left(f_{0}P\left(D_{i}|0\right)+f_{1}P\left(D_{i}|1\right)+f_{2}P\left(D_{i}|2\right)\right)-2\overset{m}{\underset{i=1}{\sum }}\text{log}P\left(D_{i}|0\right)$$

Under the null this statistic obeys a chi square distribution with two degrees of freedom [21].

If we assume Hardy-Weinberg equilibrium [22] we can estimate the frequency of the Alu deletion, *p*, on a haplotype level. Then the joint likelihood of the data given an Alu deletion is:

$$\prod_{i=1}^{m}\left({\left(1-p\right)}^{2}P\left(D_{i}|0\right)+2p\left(1-p\right)P\left(D_{i}|1\right)+p^{2}P\left(D_{i}|2\right)\right)$$

The corresponding likelihood ratio test will then obey a chi square distribution with one degree of freedom. We use the one degree of freedom test in the remainder of the paper.

### Inserted Alu identification

One of the main complications in detecting Alu polymorphisms is the fact that members of the Alu family are all highly similar. The Alu insertions which we are looking for will be similar to sequences already inserted and other sequences that also may have been inserted.

The mapping of reads not mapping to Alu regions is generally more reliable, however a number of problems may occur; the region being considered may be duplicated, or the read may be chimeric, where due to artifacts in the sequencing process two parts of the read come from different parts of the genome. This implies that not all *l *and *r *reads will be close to an actual Alu insertion. Some of the reads may also be close to Alus already discovered, but the mapping was not discovered by BWA, for a further discussion of these issues see Additional file 1. We start by finding regions that are likely to contain an insertion and then from that list we compute a probabilistic model verifying the insertion found, first for a single individual and then we extend this to multiple individuals.

#### Identifying potential inserts

As described earlier, we label Alu mates as either *l*, if their mapping to the reference genome implies that an Alu is to the left of them read or *r *if their mapping implies that an Alu is to the right of them. Each of the *l *and *r *reads then gives partial information about the location of the Alu read. Given the location of an *l *read an Alu is implied in the region from *l*_*r *_+ *Y *to *l*_*r *_+ *Y *+ *L*, where *l*_*r *_is the right endpoint of the *l *read being considered, *Y *is the distribution of the distances between paired-ends and *L *is the length of a read. Similarly, given the location of an *r *read an Alu is implied in the region from *r*_*l *_- *Y *to *r*_*l *_- *Y *- *L*, where *r*_*l *_is the left endpoint of the *r *read being considered. Some of the reads however may not be correctly mapped and should be considered errors. In particular, from the mapping of the reads to the reference genome we know the number of best mappings of the reads in question, a read that has *b *best mappings will with probability $\frac{1}{b}$ be mapped correctly. This fact means that we can in a simple manor assign weights to sequence reads, with a read having *b *best mappings getting weight $\frac{1}{b}$.

We say that an Alu position, *α, covers *an *l *read if *l*_*r *_+ *Y*_1-*ϵ *_≥ *α *and *l*_*r *_+ *Y*_*ϵ *_≤ *α *+ *L*, where *Y*_*ϵ *_and *Y*_1-*ϵ *_are defined as before. Similarly an Alu position, *α *covers an *r *read if *r*_*l *_- *Y*_1-*ϵ *_≤ *α *and *r*_*l *_- *Y*_*ϵ *_≥ *α *- *L*. For each *l *and *r *read we now want to either cover it with an Alu position or declare it as an error read, we define a constant *k *to be the relative cost between the two.

#### Problem 6

##### Alu genotyping problem

**Input **A set *L *of *l *reads and a set *R *of *r *reads.

**Output **A set *A *of Alu positions and *E *of errors.

**Objective **min | *E *| +*k *| *A *|

**Constraints **Each *l *∈ *L *and *r *∈ *R *is either in *E *or covered by an *a *∈ *A*.

We note that the most general version of this problem reduces to a set covering problem, which can be shown to be hard to even approximate [23]. However, as the reads are linearly arranged on the chromosome the sets, the problem reduces to set covering on interval graphs which can be solved in polynomial time using e.g. dynamic programming.

For our empirical evaluations we set *k *= 3, representing that at if three *l *or *r *reads are found that can be covered by a single Alu insertion we prefer to insert an Alu than to assign error labels to these reads.

##### Optimal algorithm

To search for regions likely to contain an Alu sequence we make a single pass through the genome. For each position, *p*, we sum the number of *r *reads within a window size *Y*_1-*ϵ *_to the left *p *and the number of *l *reads within a window size *Y*_1-*ϵ *_to the right of *p*.

The time complexity of the algorithm is *O*(*ncY*_1-*ϵ*_), where *n *is the length of the genome, *c *is the coverage. *w *chosen as the size of the largest Alu plus a maximum distance between paired-ends under the null distance. Regions where this indicator is above a given threshold are considered Alu regions.

#### Covering multiple individuals

One way to detect Alu insertions in multiple individuals is to pool the data into a single dataset and ignore the fact that there are multiple individuals being sequenced. This simple idea will however lack power to find infrequent Alus. A region containing multiple *l *and *r *reads in a single individual is more likely to contain an Alu than one that has a single *l *or *r *read in multiple individuals. We therefore do not want to determine an Alu unless there exist some individuals that have multiple *l *or *r *reads. We let *k*_1 _and *k*_2 _be constants, representing the cost of introducing an Alu insertion to the population and the cost of introducing an Alu insertion to each individual. We let *A *represent the set of Alus and for each Alu, *j*, we let *A_j _*be the set of individuals containing the Alu.

#### Problem 7

**Input **A set *I *of individuals. A set of *L*_*i *_of *l *reads and a set *R*_*i *_of *r *reads, for each individual *i *∈ *I*.

**Output **A set *A *of Alu positions and *E *of errors.

**Objective **min | *E *| +*k*_1 _| *A *| +*k*_2 _Σ_*j *_| *A*_*j *_|

**Constraints **Each *l *∈ *L*_*i *_and *r *∈ *R*_*i *_is either in *E *or covered by an *a *∈ *A *and *i *∈ *A*_*a*_.

We have not been able to determine the computational complexity of this problem and leave open whether or not the problem is NP-hard.

##### Heuristic algorithm

When tuning these parameters we set *k*_1 _= *k*_2 _= 2, representing that we require two sequence reads in each individual to warrant introducing a Alu insert in the population and two sequence reads to warrant introducing the Alu to the individual.

We solve this problem using a heuristic. To prune the number of regions that we need to consider we start by considering each individual at a time. In each individual we search for regions where there are at least a small number of *l *and *r *reads within the same window of size 2*Y*_1-*ϵ*_. We then merge the insert locations of two individuals if they appear to be very close to each other.

#### Genotyping of inserted Alus

Given the location of potential Alu insertions we run an algorithm similar to the one that we ran for Alus that are deleted with respect to the reference.

Until convergence

Estimate length of Alu insertion

Re-estimate positions

Insert the Alu insertion in silico in the position determined.

Apply the algorithm for deleted from reference for genotype calling.

##### Alu insertion length estimation

We assume that there is a single insertion event that occurred in all of the individuals simultaneously. For each read pair, *t*, we have given a position on the chromosome of the non-Alu read, *c*_*t*_, a position within the Alu of the Alu read *a*_*t*_, mean distance between the two, *m*_*t *_and standard deviation in distance between the two, *s*_*t*_. The means and the standard deviation are estimated from the reads of each individual independently.

Assume we know a position *p*_*Alu *_where there is an insertion. Now consider all Alu read pairs in the interval [*p*_*Alu *_- *Y*_1-*ϵ*_, *p*_*Alu *_+ *Y*_l-*ϵ*_]. Now assume that we have aligned all Alu read pairs in this interval to the same Alu, of length *l*_*Alu*_. Our model of the true length of the Alu is that it is *l*_*Alu *_+ *λ *+ *ρ*, where *λ *and *ρ *are constants, which can be either positive or negative. *λ *represents a left offset in the length of the Alu and *ρ *represents a right offset in the length of the Alu.

We now estimate *λ *and *ρ *seperately. We start by considering all reads pairs with the non-Alu read in [*p*_*Alu *_- *Y*_1-*ϵ*_, *p*_*Alu*_] and use these to estimate *λ*. Let *d*_*t *_= *p*_*Alu *_- *c*_*t*_, then the estimate of *λ *from *t *is *λ*^*t *^= *m*_*t *_- *d*_*t *_- *a*_*t*_, with standard deviation *s*_*t*_. When considering multiple reads the maximum likelihood estimate of *λ *is then:

$$\lambda =\frac{\sum_{t}\frac{\lambda_{t}}{s_{t}^{2}}}{\sum_{t}\frac{1}{s_{t}^{2}}}$$

Similarly we get an estimate for *ρ *by setting $a_{t}^{-}=l_{Alu}-a_{t}$. We now consider all Alu read pairs with a non Alu read in the interval [*p*_*Alu*_, *p*_*Alu *_+ *Y*_l-*ϵ*_] and use these to get an estimate of *ρ*. Let *d*_*t *_= *c*_*t *_- *p*_*Alu*_, then the estimate of *ρ *from *t *is $\rho_{t}=m_{t}-d_{t}-a_{t}^{-}$ with standard deviation *s*_*t*_. When considering multiple reads the maximum likelihood estimate of *ρ *is then:

$$\rho =\frac{\sum_{t}\frac{\rho_{t}}{s_{t}^{2}}}{\sum_{t}\frac{1}{s_{t}^{2}}}$$

#### Alu insert position reestimation

Each read gives an estimate of the location of the inserted Alu. A joint estimate is determined from all of the reads in a given region. This is done in the same manor as described above, where we isolate *p*_*Alu *_from the equations instead of *λ *and *ρ*.

##### In silico insertion and deleted algorithm

Once the location of the Alu insertion and the length of the Alu is determined a new sequence is constructed containing the Alu at the inserted location. Following the construction of this new sequence a graph, identical to the one described for Alus deleted with respect to the reference, containing the location of the reads in the interval is constructed as before.

The in silico constructed genomic sequence now contains the Alu that we previously considered to be inserted. The Alu sequence is therefore deleted with respect to this sequence and we can apply the same algorithm as before.

## Results

We run our experiments on simulated data and on data from the 1000 genomes project.

### Simulated data

We benchmark our algorithms on simulated data. We downloaded chromosome 22 of build 37 of the human genome, as well as the RepeatMasker track to identify Alu sequences in the build. We downloaded a database of Alu sequences from RepBase [24]. We selected four Alu sequences known to be active in humans; AluYa5,AluYb8,AluYb9,AluYk13; and AluJo, a sequence not known to be active. At each location the Alu sequences were mutated independently with a 3% uniform mutation frequency. Each of the five Alus was inserted at ten different locations, for a total of 50 Alus inserted. We inserted the Alus into 100 different chromosomes. At each location we used one of ten different frequencies of insertion; 2, 4, 5, 10, 20, 80, 90, 94, 96, 98%. As each Alu was inserted into a different number of chromosomes depending on their frequency, each chromosome contained on average 25 Alu insertions, ranging from 21 to 33 Alus inserted into each chromosome.

The 100 chromosomes where then paired to construct 50 diploid individuals, with each individual containing on average 50 Alu insertions. The Alu insert locations were chosen randomly on the chromosome, with the constraint that no Alu was added within *Y*_1-*ϵ *_basepairs of another Alu and no more than 1% of basepairs are annotated N in a 2*Y*_1-*ϵ *_basepair window surrounding the introduced Alu. This allows us to focus our results only on Alu insertions that are distant from other Alus and is not meant to representative of the process in which Alu's are inserted. Reads were simulated using the program SimSeq [25]. Reads were simulated independently for each chromosome, with an average of 5*x *coverage per chromosome or 10*x *coverage per individual. In our experiments 97% of all reads not mapping to Alu regions mapped uniquely to the genome, using BWA. We simulated our data with both with no error and with 2% error.

#### Alu insertion

The set of individuals were selected to have similar coverage and being genotyped under similar conditions. We benchmark our Alu insertion identification algorithm by considering the mapping of the reads of the simulated individuals to the reference genome, results are shown in Table 1.

**Table 1:** Alus inserted with respect to the reference

	Expected	Found(%)
Error free	1512	1483 (98.1%)

2% error	1512	1446(95.6%)

Number of Alus found inserted with respect to the reference in simulated genotype data.

We ran our insertion algorithm on each individual independently. When tuning our algorithms to find no false positives we find 96.4% of all Alus inserted. The false negatives are mostly from individuals that are heterozygote for the insertion and are mostly when there is other surrounding variation.

#### Alu deletion

We benchmark our Alu deletion identification algorithm by considering the mapping of the reads of the simulated individuals to a simulated individual that contains all the Alus, results are shown in Table 2. When tuning our algorithms to find no false positives we find 97.7% of all Alus deleted. We find deleted Alu's in 1390 of the 1422 locations known to contain an Alu.

**Table 2:** Alus deleted with respect to the reference

	Expected	Found(%)
Error free	1422	1390(97.7%)

2% error	1422	1385(97.4%)

Number of Alus found deleted with respect to the reference in simulated genotype data.

In Additional file 1 we investigate the effects of higher error rate on our algorithm.

### Verification on triad data

We investigated whether the the deletions that we detected were transmitted to the children. We simulated fifty trios where we independently simulated two chromosomes with randomly inserted Alus for each parent. We then randomly selected one chromosome from each parent to use for the child. We found very high concordance between parent and the child, as shown in Table 3.

**Table 3:** Trio results

	Found in child	Matches parents
Homozygote deleted	997	997 (100%)

Heterozygote	368	362 (98.4%)

The number of deletions found in child that were also found in a consistent manor in its parents. The first line shows when the child is homozygote for the deletion. The second line shows the results when the child carries only a single copy of the deletion.

### 1000 genomes

We run our experiments on twenty individuals from LWK: Luhya in Webuye, Kenya population of the 1000 genomes project [6,26,27].

We find an average of 1418 Alus that are deleted with respect to the reference. This corresponds to a rate of approximately $\frac{1}{1000}$ Alus in the human genome being deleted with respect to the reference, a rate comparable to the SNP polymorphism rate. A table showing the number of Alus deleted with respect to the reference in each individual in the LWK population is shown in Additional file 1.

We find an average of 5990 Alus that are inserted with respect to the reference. A table showing the number of inserted Alus in each individual in the is shown in Additional file 1.

dbRIP [28] is database containing 2083 Alus known to be polymorphic in the human population. On average each one of our individuals contains 280 of the Alus represented in dbRIP.

Stewart et al. [29] found a total of 1730 Alus that were deleted with respect to the reference and 4499 Alus that were inserted with respect to the reference when, considering a subset of the 1000 genomes population. The individuals considered by Stewart et al. were not the same as the ones considered by us. We note that this number is lower than we are finding, we have not investigated the source of this difference and it may be due to the fact that our method is more sensitive or gives more false positives. When comparing a single individual to the set of deletions found by Stewart et al we find that on average 73.4% of the deleted that we find were found in some of the individuals studied by Stewart et al. We find that 7.2% of the inserted Alus that we find are found in some of the individuals studied by Stewart et al. The high concordance for the deleted case is promising. The comparatively lower concordance with the inserted Alus may be due to the fact that our algorithm has a high false positive rate, but also may be due to the fact that Alu insertions are of low frequency and the population that we study is distantly related from the population studied by Stewart et al.

When we compare the deleted Alus of two individuals we found that 61.5% of the deletions found in one individual are also found in another individual. For inserted Alus this number is 15.6%. The reason for this difference is the fact that Alus generally have a low frequency, the deleted Alus are generally the ones that have been inserted into the reference genome and hence they will not be present in a large number of the other individuals, while the inserted have only been inserted into a subset of the population.

#### Timing

We ran our computations on desktop machine using a single 3.06 GHz Intel i5 processor. On average each individual of the 1000 genomes data took 1hr and 44 minutes to analyze regions that are deleted with respect to the reference and 2hrs and 1 minute to analyze regions that are inserted with respect to the reference.

### Alu families

We investigate which Alu families are deleted. We estimate the Alu family from the repeat masker annotations (cf. Table 4). Using these annotations 82.15% of the deletions are found to belong to the AluY family. This family is believed to be the family most polymorphic in humans [15]. We also find that 11.48% of the deletions that we find belong to the AluS family and 6.38% belong to the AluJ family.

**Table 4:** Estimated Alu families

	Total
AluY	22660(82,15%)

AluS	3167(11,48%)

AluJ	1758(6,38%)

Estimated Alu families of Alus deleted with respect to the reference genome using 1000 Genomes data.

## Conclusions

A number of improvements can be made to the the algorithm that we have presented. Broken reads, those where one part maps to the reference genome and one part maps to an insertion or where one part maps to one side of an deletion and one part to the other, can be used to improve the algorithms described here. In our algorithm we study only the single best mapping of each sequence read. An alternative would be to study multiple mapping of reads to the reference genome. We will attempt to explore such solutions, however our experimental results suggests that this will provide little gain for most regions of the genome with considerable added algorithmic complexity. Our future goals are to extend the methods developed here to find other types of structural variations.

## List of abbreviations

SNP: single nucleotide polymorphism; DNA: deoxyribonucleic acid; RNA: ribonucleic acid; LINE: long interspersed elements; SINE: short interspersed elements; GWAS: genomewide association studies; LWK: Luhya in Webuye, Kenya.

## Competing interests

The authors declare that they have no competing interests.

## Authors' contributions

JIS implemented the software. BVH and JIS ran the experiments. BVH and JIS developed the algorithms. BVH wrote the first draft of the paper. BVH and JIS contributed to writing the final version of the paper.

## Supplementary Material

Additional file 1 **Supplementary material contains a more detailed description of our methods, additional simulation results and results on the 1000 genomes data**. Click here for file
