# Supplementary material for: PAIR: polymorphic Alu insertion recognition
Source: BMC Bioinformatics. 2012 Apr 19;13(Suppl 6):S7. doi: 10.1186/1471-2105-13-S6-S7 (PMC3358660; doi:10.1186/1471-2105-13-S6-S7)
Supplement: Additional file 1 — Supplementary material contains a more detailed description of our methods, additional simulation results and results on the 1000 genomes data. [file 1471-2105-13-S6-S7-S1.pdf]

Additional file 1

Estimating Y

For each chromosome we consider all reads that map in a proper pair on that chromosome. We then compute average and standard deviation in the distance between read pairs. We then approximate Y with a normal distribution.

l and r read reassignment

Some of the reads that we find can be removed before further analysis. For all the l and r reads we start by aligning the Alu read to the interval where we expect the read to be mapped based on it's mapped ($[p_m + Y_\epsilon, p_m + Y_{1-\epsilon}]$, where p_m is the position of the mate). If a good match is found in that interval we assume that this is the correct mapping which was not correctly identified by BWA. We are able to search for these patterns quickly using interval trees.

Deleted in 1000 genomes

Individual	Deletions found
NA19383	1554
NA19399	1519
NA19428	1340
NA19391	1325
NA19403	1302
NA19395	1493
NA19381	1170
NA19359	1375
NA19397	1446
NA19375	1465
NA19396	1480
NA19373	1458
NA19379	1431
NA19401	1386
NA19374	1344
NA19390	1542
NA19371	1457
NA19398	1473
NA19376	1399
NA19355	1401

Figure 1: Table shows the number of Alus found that were deleted with respect to the reference genome in the 1000 genomes population

Inserted in 1000 genomes

Individual	Insertions found
NA19383	8071
NA19399	5247
NA19428	6157
NA19391	5387
NA19403	5805
NA19395	5383
NA19381	5059
NA19359	5724
NA19397	6015
NA19375	4588
NA19396	7738
NA19373	4586
NA19379	5810
NA19401	5930
NA19374	5018
NA19390	6819
NA19371	8642
NA19398	7078
NA19376	5048
NA19355	5705

Figure 2: Table shows the number of Alus found that were deleted with respect to the reference genome in the 1000 genomes population

Increasing the error rate

We investigate how increasing the error rate would change our results. We observe that increasing the error rate to 4% has a small effect on our prediction rate while increasing to 6% has quite a negative effect.

Individual	Deleted Alus	Deleted Alus Found(%)
Error free	1422	1390(97.7%)
2% error	1422	1385(97.4%)
4% error	1422	1330(93.5%)
6% error	1422	1120(78.7%)

Figure 3: Table shows the affect of higher error rate on our algorithms ability to find Alu deleted with respect to the reference

Individual	Inserted Alus	Inserted Alus Found(%)
Error free	1512	1483 (98.1%)
2% error	1512	1446(95.6%)
4% error	1512	1342 (88.8%)
6% error	1512	1081(71.5%)

Figure 4: Table shows the affect of higher error rate on our algorithms ability to find Alu deleted with respect to the reference
